# Tris(1,10-phenanthroline)nickel(II) dichromate tetra­hydrate

**DOI:** 10.1107/S1600536810000802

**Published:** 2010-01-13

**Authors:** Hai-Xing Liu

**Affiliations:** a College of Chemistry and Chemical Engineering, Microscale Science Institute, Weifang University, Weifang 261061, People’s Republic of China

## Abstract

The asymmetric unit of the title compound, [Ni(C_12_H_8_N_2_)_3_][Cr_2_O_7_]·4H_2_O, contains one cation, one anion and four water mol­ecules, three of which are disordered over two sites with equal occupancies. In the cation, the metal centre is coordinated by six N atoms from three 1,10-phenanthroline ligands in a distorted octa­hedral geometry. The [Cr_2_O_7_]^2−^ anion exhibits a staggered conformation. The crystal packing is stabilized by inter­molecular O—H⋯O hydrogen bonds and π–π inter­actions, evidenced by short distances of 3.531 (5) Å between the centroids of aromatic rings.

## Related literature

For related structures, see: Ejsmont *et al.* (2002 ▶); Suescun *et al.* (1999 ▶); Wang *et al.* (2007 ▶); Wiehl *et al.* (2008 ▶).
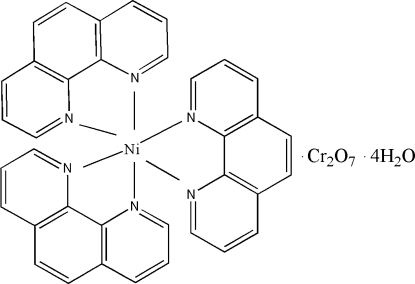


## Experimental

### 

#### Crystal data

[Ni(C_12_H_8_N_2_)_3_][Cr_2_O_7_]·4H_2_O
*M* *_r_* = 887.39
Monoclinic, 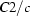

*a* = 26.899 (2) Å
*b* = 17.8121 (16) Å
*c* = 17.3656 (18) Å
β = 105.274 (2)°
*V* = 8026.5 (13) Å^3^
*Z* = 8
Mo *K*α radiation
μ = 1.06 mm^−1^
*T* = 298 K
0.14 × 0.12 × 0.11 mm

#### Data collection

Bruker APEXII CCD area-detector diffractometer
Absorption correction: multi-scan (*SADABS*; Sheldrick, 1996 ▶) *T*_min_ = 0.866, *T*_max_ = 0.892
21223 measured reflections
7229 independent reflections
2790 reflections with *I* > 2σ(*I*)
*R*_int_ = 0.129

#### Refinement

*R*[*F* ^2^ > 2σ(*F* ^2^)] = 0.077
*wR*(*F* ^2^) = 0.218
*S* = 1.06
7229 reflections
532 parameters
H-atom parameters constrained
Δρ_max_ = 0.80 e Å^−3^
Δρ_min_ = −0.37 e Å^−3^

### 

Data collection: *APEX2* (Bruker, 2000 ▶); cell refinement: *SAINT* (Bruker, 2000 ▶); data reduction: *SAINT*; program(s) used to solve structure: *SHELXS97* (Sheldrick, 2008 ▶); program(s) used to refine structure: *SHELXL97* (Sheldrick, 2008 ▶); molecular graphics: *SHELXTL* (Sheldrick, 2008 ▶); software used to prepare material for publication: *SHELXTL*.

## Supplementary Material

Crystal structure: contains datablocks global, I. DOI: 10.1107/S1600536810000802/cv2680sup1.cif

Structure factors: contains datablocks I. DOI: 10.1107/S1600536810000802/cv2680Isup2.hkl

Additional supplementary materials:  crystallographic information; 3D view; checkCIF report

## Figures and Tables

**Table 1:** Hydrogen-bond geometry (Å, °)

*D*—H⋯*A*	*D*—H	H⋯*A*	*D*⋯*A*	*D*—H⋯*A*
O14—H14*D*⋯O10^i^	0.85	2.06	2.91 (3)	177
O14—H14*C*⋯O10^ii^	0.85	1.75	2.60 (3)	176
O13—H13*D*⋯O14	0.85	1.76	2.61 (3)	179
O13—H13*C*⋯O2^iii^	0.85	1.98	2.83 (2)	176
O12—H12*D*⋯O9^iv^	0.85	2.16	2.99 (2)	165
O12—H12*C*⋯O14	0.85	2.11	2.93 (3)	165
O10—H10*D*⋯O11	0.85	1.93	2.78 (3)	171
O10—H10*C*⋯O1	0.85	1.91	2.751 (17)	172
O9—H9*A*⋯O12^v^	0.85	2.24	2.99 (2)	147
O9—H9*D*⋯O9^vi^	0.85	1.94	2.78 (3)	176
O9—H9*C*⋯O8	0.85	2.11	2.962 (17)	177
O8—H8*D*⋯O11^vii^	0.85	1.92	2.76 (2)	167
O8—H8*C*⋯O6	0.85	2.00	2.836 (11)	168

Symmetry codes: (i) 
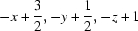
; (ii) 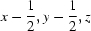; (iii) 
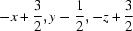
; (iv) 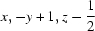; (v) 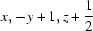; (vi) 
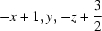
; (vii) 
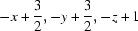
.

**Fig. 1.**
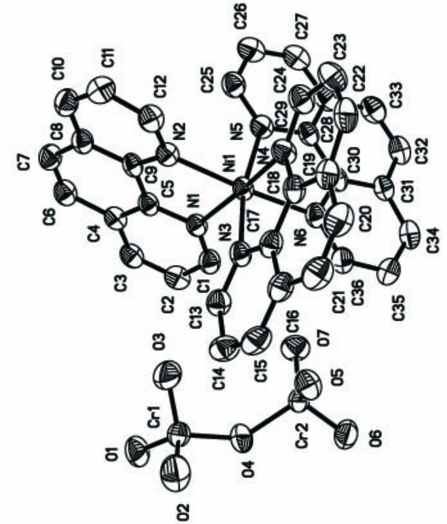
The molecular structure of the title compound with the atom-labeling scheme. Displacement ellipsoids are drawn at the 30% probability level. The lattice water molecules and H atoms are omitted for clarity.

## supplementary crystallographic information

### Comment

Metal complexes containing the chromate(VI) or dichromate(VI) anions, CrO_4_^2-^ or Cr_2_O_7_^2-^, attract attention owing to their various properties (Ejsmont *et al.*, 2002). 1,10-Phenanthroline (phen), which is the parent of an important class of chelating agents, has been widely used in the construction of supramolecular architectures. Some Nickel phenanthroline complexes have been synthesized and reported (Suescun *et al.*, 1999; Wang *et al.*, 2007; Wiehl *et al.*, 2008). As a continuation of these studies, I now report the crystal structure of the title complex.

The title structure (Fig. 1) is build up of one Ni atom, three coordination phenanthroline ligand, dichromate and four free water molecules. Ni center is coordinated with six N atoms from three 1,10-phenanthroline ligands, presenting a distorted octahedral geometry. The Ni—N and Cr—O bond lengths are in the ranges 2.064 (7)–2.115 (7) and 1.583 (7)–1.786 (6) Å, respectively. The bond angles O—Cr—O, N—Ni—N are in the ranges of 107.1 (3)–111.4 (4) and 78.6 (3)–168.9 (3) °, respectively. The mean Cr—O bond lengths and O—Cr—O bond angles are similar to the reported earlier (Ejsmont *et al.*, 2002).

The crystal packing is stabilized by intermolecular O—H···O hydrogen bonds (Table 1) and π-π interactions evidenced by short distance of 3.531 (5) Å between the centroids of aromatic rings.

### Experimental

All commercially obtained reagent-grade chemicals were used without further purication. A mixture of NiCl_2_ (0.1 mmol, 0.014 g), 1,10-phenanthroline (0.1 mmol, 0.018 g), K_2_CrO_4_ (0.1 mmol, 0.019 g) and H_3_BO_3_ (0.1 mmol, 0.006 g) were added into 20 ml water with 20%(*v*/*v*) ethanol and heated for 12 h at 140 °. The solution was obtained by filtration after cooling the reaction to room temperature. Yellow block single crystals suitable for X-ray measurements were obtained after a few weeks.

### Refinement

All H atoms were geometrically positioned (C—H 0.93 Å, O—H 0.85 Å) and refined using a riding model, with *U*_iso_ (H) =1.2–1.5 *U*_eq_ of the parent atom.

### Crystal data

**Table:**

[Ni(C_12_H_8_N_2_)_3_][Cr_2_O_7_]·4H_2_O	*F*(000) = 3632
*M**_r_* = 887.39	*D*_x_ = 1.469 Mg m^−^^3^
Monoclinic, *C*2/*c*	Mo *K*α radiation, λ = 0.71073 Å
*a* = 26.899 (2) Å	Cell parameters from 1859 reflections
*b* = 17.8121 (16) Å	θ = 2.5–25.1°
*c* = 17.3656 (18) Å	µ = 1.06 mm^−^^1^
β = 105.274 (2)°	*T* = 298 K
*V* = 8026.5 (13) Å^3^	Block, yellow
*Z* = 8	0.14 × 0.12 × 0.11 mm

### Data collection

**Table:**

Bruker APEXII CCD area-detector diffractometer	7229 independent reflections
Radiation source: fine-focus sealed tube	2790 reflections with *I* > 2σ(*I*)
graphite	*R*_int_ = 0.129
φ and ω scans	θ_max_ = 25.2°, θ_min_ = 1.4°
Absorption correction: multi-scan (*SADABS*; Sheldrick, 1996)	*h* = −32→31
*T*_min_ = 0.866, *T*_max_ = 0.892	*k* = −17→21
21223 measured reflections	*l* = −20→20

### Refinement

**Table:**

Refinement on *F*^2^	Primary atom site location: structure-invariant direct methods
Least-squares matrix: full	Secondary atom site location: difference Fourier map
*R*[*F*^2^ > 2σ(*F*^2^)] = 0.077	Hydrogen site location: inferred from neighbouring sites
*wR*(*F*^2^) = 0.218	H-atom parameters constrained
*S* = 1.06	*w* = 1/[σ^2^(*F*_o_^2^) + (0.0732*P*)^2^] where *P* = (*F*_o_^2^ + 2*F*_c_^2^)/3
7229 reflections	(Δ/σ)_max_ = 0.001
532 parameters	Δρ_max_ = 0.80 e Å^−^^3^
0 restraints	Δρ_min_ = −0.37 e Å^−^^3^

### Special details

**Table:**

Geometry. All e.s.d.'s (except the e.s.d. in the dihedral angle between two l.s. planes) are estimated using the full covariance matrix. The cell e.s.d.'s are taken into account individually in the estimation of e.s.d.'s in distances, angles and torsion angles; correlations between e.s.d.'s in cell parameters are only used when they are defined by crystal symmetry. An approximate (isotropic) treatment of cell e.s.d.'s is used for estimating e.s.d.'s involving l.s. planes.
Refinement. Refinement of *F*^2^ against ALL reflections. The weighted *R*-factor *wR* and goodness of fit *S* are based on *F*^2^, conventional *R*-factors *R* are based on *F*, with *F* set to zero for negative *F*^2^. The threshold expression of *F*^2^ > σ(*F*^2^) is used only for calculating *R*-factors(gt) *etc*. and is not relevant to the choice of reflections for refinement. *R*-factors based on *F*^2^ are statistically about twice as large as those based on *F*, and *R*- factors based on ALL data will be even larger.

### Fractional atomic coordinates and isotropic or equivalent isotropic displacement parameters (Å^2^)

**Table:**

	*x*	*y*	*z*	*U*_iso_*/*U*_eq_	Occ. (<1)
Cr1	0.82179 (6)	0.55759 (10)	0.64402 (9)	0.0650 (5)	
Cr2	0.72271 (6)	0.63263 (9)	0.68533 (9)	0.0587 (5)	
Ni1	0.63211 (4)	0.33221 (7)	0.65097 (6)	0.0490 (4)	
N1	0.6607 (3)	0.3733 (5)	0.5584 (4)	0.0494 (18)	
N2	0.6566 (3)	0.2337 (4)	0.6091 (4)	0.0509 (19)	
N3	0.6977 (3)	0.3555 (4)	0.7406 (4)	0.054 (2)	
N4	0.6129 (3)	0.2824 (4)	0.7479 (4)	0.060 (2)	
N5	0.5576 (3)	0.3205 (5)	0.5728 (4)	0.058 (2)	
N6	0.5977 (3)	0.4336 (4)	0.6700 (4)	0.056 (2)	
O1	0.8457 (3)	0.5751 (4)	0.5699 (4)	0.090 (2)	
O2	0.8657 (3)	0.5494 (5)	0.7250 (4)	0.118 (3)	
O3	0.7903 (3)	0.4816 (4)	0.6280 (5)	0.110 (3)	
O4	0.7807 (2)	0.6314 (4)	0.6536 (4)	0.078 (2)	
O5	0.7274 (2)	0.5758 (4)	0.7583 (4)	0.079 (2)	
O6	0.7148 (3)	0.7165 (4)	0.7117 (4)	0.092 (2)	
O7	0.6742 (2)	0.6079 (4)	0.6147 (4)	0.084 (2)	
O8	0.6132 (3)	0.7723 (5)	0.6890 (6)	0.156 (4)	
H8C	0.6449	0.7623	0.6945	0.187*	
H8D	0.6085	0.8189	0.6791	0.187*	
O9	0.5502 (5)	0.7309 (8)	0.7989 (9)	0.099 (5)	0.50
H9C	0.5693	0.7427	0.7689	0.119*	0.50
H9D	0.5191	0.7305	0.7708	0.119*	0.25
H9A	0.5537	0.7627	0.8365	0.119*	0.25
O10	0.9278 (6)	0.5528 (10)	0.5059 (12)	0.149 (7)	0.50
H10C	0.9007	0.5608	0.5210	0.179*	0.50
H10D	0.9213	0.5619	0.4562	0.179*	0.50
O11	0.9181 (7)	0.5812 (11)	0.3454 (13)	0.161 (8)	0.50
H11C	0.8930	0.5622	0.3105	0.193*	0.50
H11D	0.9429	0.5500	0.3534	0.193*	0.50
O12	0.5385 (6)	0.1208 (10)	0.3729 (11)	0.142 (7)	0.50
H12C	0.5353	0.1194	0.4202	0.171*	0.50
H12D	0.5418	0.1663	0.3602	0.171*	0.25
H12A	0.5126	0.1007	0.3405	0.171*	0.25
O13	0.5318 (8)	0.0721 (11)	0.6833 (14)	0.187 (9)	0.50
H13C	0.5631	0.0654	0.7087	0.225*	0.50
H13D	0.5301	0.0758	0.6339	0.225*	0.50
O14	0.5251 (8)	0.0848 (12)	0.5311 (14)	0.189 (9)	0.50
H14C	0.4929	0.0762	0.5210	0.227*	0.50
H14D	0.5399	0.0454	0.5205	0.227*	0.50
C1	0.6671 (3)	0.4424 (6)	0.5391 (6)	0.059 (3)	
H1	0.6609	0.4805	0.5720	0.071*	
C2	0.6829 (3)	0.4620 (6)	0.4709 (6)	0.067 (3)	
H2	0.6870	0.5121	0.4587	0.080*	
C3	0.6920 (3)	0.4070 (7)	0.4237 (6)	0.063 (3)	
H3	0.7024	0.4190	0.3782	0.076*	
C4	0.6862 (3)	0.3333 (6)	0.4419 (5)	0.055 (2)	
C5	0.6716 (3)	0.3183 (6)	0.5123 (5)	0.051 (2)	
C6	0.6965 (3)	0.2689 (7)	0.3962 (6)	0.066 (3)	
H6	0.7062	0.2769	0.3492	0.079*	
C7	0.6924 (4)	0.2002 (7)	0.4203 (6)	0.067 (3)	
H7	0.6985	0.1605	0.3892	0.080*	
C8	0.6788 (3)	0.1839 (6)	0.4934 (6)	0.057 (3)	
C9	0.6681 (3)	0.2430 (6)	0.5386 (5)	0.050 (2)	
C10	0.6762 (3)	0.1114 (6)	0.5231 (6)	0.069 (3)	
H10	0.6821	0.0700	0.4941	0.083*	
C11	0.6650 (4)	0.1016 (6)	0.5956 (6)	0.070 (3)	
H11	0.6634	0.0540	0.6166	0.085*	
C12	0.6564 (3)	0.1647 (7)	0.6352 (6)	0.064 (3)	
H12	0.6498	0.1581	0.6848	0.077*	
C13	0.7396 (4)	0.3902 (5)	0.7369 (6)	0.062 (3)	
H13	0.7433	0.4036	0.6869	0.074*	
C14	0.7796 (4)	0.4087 (6)	0.8048 (6)	0.073 (3)	
H14	0.8093	0.4329	0.8001	0.088*	
C15	0.7732 (4)	0.3897 (6)	0.8774 (6)	0.073 (3)	
H15	0.7986	0.4026	0.9232	0.088*	
C16	0.7301 (4)	0.3520 (6)	0.8846 (6)	0.065 (3)	
C17	0.6925 (4)	0.3355 (5)	0.8137 (6)	0.058 (2)	
C18	0.6481 (4)	0.2968 (5)	0.8176 (6)	0.056 (2)	
C19	0.6411 (4)	0.2738 (6)	0.8903 (6)	0.063 (3)	
C20	0.6802 (5)	0.2927 (6)	0.9619 (6)	0.076 (3)	
H20	0.6763	0.2775	1.0112	0.092*	
C21	0.7208 (5)	0.3306 (6)	0.9587 (6)	0.081 (3)	
H21	0.7445	0.3442	1.0061	0.097*	
C22	0.5978 (5)	0.2301 (6)	0.8885 (6)	0.075 (3)	
H22	0.5927	0.2126	0.9363	0.090*	
C23	0.5630 (4)	0.2125 (6)	0.8191 (6)	0.075 (3)	
H23	0.5345	0.1826	0.8180	0.090*	
C24	0.5722 (4)	0.2416 (6)	0.7488 (6)	0.071 (3)	
H24	0.5482	0.2315	0.7005	0.085*	
C25	0.5391 (4)	0.2646 (6)	0.5224 (6)	0.066 (3)	
H25	0.5603	0.2240	0.5196	0.079*	
C26	0.4877 (4)	0.2658 (7)	0.4726 (6)	0.074 (3)	
H26	0.4758	0.2270	0.4367	0.089*	
C27	0.4568 (4)	0.3230 (7)	0.4781 (6)	0.070 (3)	
H27	0.4230	0.3238	0.4463	0.084*	
C28	0.4749 (4)	0.3814 (6)	0.5310 (6)	0.060 (3)	
C29	0.5256 (4)	0.3790 (6)	0.5769 (5)	0.053 (2)	
C30	0.5460 (4)	0.4384 (6)	0.6284 (5)	0.054 (2)	
C31	0.5155 (4)	0.5002 (6)	0.6345 (6)	0.060 (3)	
C32	0.4630 (4)	0.4995 (7)	0.5871 (7)	0.073 (3)	
H32	0.4417	0.5397	0.5908	0.088*	
C33	0.4445 (4)	0.4443 (7)	0.5394 (7)	0.075 (3)	
H33	0.4103	0.4462	0.5098	0.090*	
C34	0.5386 (4)	0.5586 (6)	0.6846 (6)	0.070 (3)	
H34	0.5193	0.6003	0.6914	0.084*	
C35	0.5904 (4)	0.5541 (6)	0.7243 (6)	0.072 (3)	
H35	0.6066	0.5936	0.7561	0.086*	
C36	0.6175 (4)	0.4912 (6)	0.7162 (6)	0.063 (3)	
H36	0.6520	0.4886	0.7448	0.076*	

### Atomic displacement parameters (Å^2^)

**Table:**

	*U*^11^	*U*^22^	*U*^33^	*U*^12^	*U*^13^	*U*^23^
Cr1	0.0721 (11)	0.0780 (13)	0.0503 (10)	−0.0063 (10)	0.0256 (9)	−0.0105 (9)
Cr2	0.0673 (10)	0.0675 (11)	0.0454 (9)	−0.0016 (9)	0.0223 (8)	−0.0058 (8)
Ni1	0.0473 (7)	0.0651 (8)	0.0354 (6)	0.0018 (6)	0.0123 (5)	−0.0019 (6)
N1	0.051 (4)	0.058 (5)	0.042 (5)	0.001 (4)	0.016 (4)	0.003 (4)
N2	0.054 (5)	0.058 (6)	0.041 (5)	0.000 (4)	0.013 (4)	0.003 (4)
N3	0.053 (5)	0.067 (6)	0.043 (5)	0.005 (4)	0.016 (4)	−0.002 (4)
N4	0.061 (5)	0.076 (6)	0.046 (5)	−0.005 (5)	0.019 (4)	0.002 (4)
N5	0.057 (5)	0.073 (6)	0.043 (5)	−0.005 (5)	0.013 (4)	−0.003 (4)
N6	0.058 (5)	0.069 (6)	0.043 (5)	0.005 (4)	0.019 (4)	−0.003 (4)
O1	0.092 (5)	0.115 (6)	0.077 (5)	0.004 (4)	0.049 (4)	−0.003 (4)
O2	0.098 (6)	0.171 (8)	0.073 (5)	0.031 (6)	0.004 (5)	−0.021 (5)
O3	0.133 (7)	0.097 (6)	0.124 (7)	−0.032 (5)	0.075 (6)	−0.032 (5)
O4	0.078 (4)	0.090 (5)	0.077 (5)	0.006 (4)	0.040 (4)	−0.002 (4)
O5	0.084 (5)	0.097 (5)	0.063 (4)	0.005 (4)	0.031 (4)	0.014 (4)
O6	0.110 (6)	0.071 (5)	0.109 (6)	0.002 (4)	0.055 (5)	−0.014 (4)
O7	0.074 (5)	0.104 (6)	0.063 (5)	−0.007 (4)	0.001 (4)	−0.005 (4)
O8	0.119 (7)	0.154 (9)	0.176 (10)	0.007 (6)	0.006 (7)	−0.031 (7)
O9	0.088 (10)	0.107 (12)	0.106 (12)	0.001 (9)	0.032 (9)	−0.014 (10)
O10	0.122 (14)	0.174 (19)	0.18 (2)	−0.008 (13)	0.084 (14)	−0.032 (15)
O11	0.146 (16)	0.156 (18)	0.18 (2)	0.018 (14)	0.048 (15)	−0.010 (15)
O12	0.145 (15)	0.133 (16)	0.143 (17)	0.005 (13)	0.029 (13)	−0.005 (13)
O13	0.161 (19)	0.18 (2)	0.21 (2)	0.002 (16)	0.024 (18)	−0.033 (17)
O14	0.167 (19)	0.17 (2)	0.21 (2)	−0.006 (16)	0.019 (18)	0.003 (18)
C1	0.060 (6)	0.069 (8)	0.049 (6)	0.005 (6)	0.017 (5)	−0.003 (6)
C2	0.064 (7)	0.076 (8)	0.060 (7)	−0.004 (6)	0.013 (6)	0.010 (6)
C3	0.063 (7)	0.081 (8)	0.049 (6)	−0.002 (6)	0.023 (5)	0.010 (6)
C4	0.049 (5)	0.071 (7)	0.044 (6)	0.001 (5)	0.011 (5)	0.006 (6)
C5	0.046 (5)	0.070 (7)	0.040 (5)	0.002 (5)	0.014 (4)	−0.003 (5)
C6	0.065 (7)	0.089 (9)	0.048 (6)	0.002 (6)	0.020 (5)	−0.002 (6)
C7	0.073 (7)	0.079 (8)	0.051 (7)	0.000 (6)	0.021 (5)	−0.014 (6)
C8	0.054 (6)	0.068 (8)	0.050 (6)	0.004 (5)	0.017 (5)	−0.003 (6)
C9	0.048 (6)	0.064 (7)	0.043 (6)	0.003 (5)	0.020 (5)	0.002 (5)
C10	0.066 (7)	0.075 (8)	0.063 (7)	0.008 (6)	0.012 (6)	−0.013 (6)
C11	0.077 (7)	0.066 (8)	0.065 (7)	0.002 (6)	0.011 (6)	0.005 (6)
C12	0.067 (6)	0.075 (8)	0.050 (6)	0.003 (6)	0.017 (5)	0.004 (6)
C13	0.064 (7)	0.069 (7)	0.051 (6)	0.004 (6)	0.013 (6)	−0.003 (5)
C14	0.069 (7)	0.075 (8)	0.065 (8)	−0.005 (6)	−0.001 (6)	−0.006 (6)
C15	0.081 (8)	0.072 (8)	0.053 (7)	0.005 (7)	−0.007 (6)	−0.009 (6)
C16	0.078 (7)	0.065 (7)	0.049 (7)	0.009 (6)	0.012 (6)	−0.004 (5)
C17	0.063 (6)	0.064 (7)	0.043 (6)	0.010 (6)	0.009 (5)	−0.006 (5)
C18	0.065 (7)	0.067 (7)	0.041 (6)	0.010 (6)	0.020 (5)	0.002 (5)
C19	0.074 (7)	0.066 (7)	0.053 (7)	0.012 (6)	0.023 (6)	0.000 (6)
C20	0.096 (9)	0.083 (8)	0.048 (7)	0.009 (7)	0.015 (7)	0.001 (6)
C21	0.096 (9)	0.084 (9)	0.054 (7)	0.007 (7)	0.006 (7)	−0.004 (6)
C22	0.091 (8)	0.087 (9)	0.057 (7)	0.020 (7)	0.037 (7)	0.005 (6)
C23	0.070 (7)	0.090 (8)	0.071 (8)	0.002 (6)	0.029 (7)	0.020 (7)
C24	0.070 (7)	0.088 (8)	0.057 (7)	0.002 (6)	0.023 (6)	0.004 (6)
C25	0.065 (7)	0.079 (8)	0.057 (7)	0.001 (6)	0.020 (6)	0.000 (6)
C26	0.069 (8)	0.092 (9)	0.061 (7)	−0.013 (7)	0.014 (6)	0.004 (6)
C27	0.058 (7)	0.092 (9)	0.057 (7)	−0.003 (7)	0.010 (6)	0.016 (7)
C28	0.053 (7)	0.075 (8)	0.053 (7)	0.000 (6)	0.019 (6)	0.009 (6)
C29	0.051 (6)	0.069 (8)	0.043 (6)	0.003 (6)	0.020 (5)	0.006 (5)
C30	0.056 (7)	0.067 (7)	0.045 (6)	0.004 (6)	0.024 (5)	0.006 (5)
C31	0.055 (7)	0.074 (8)	0.055 (6)	0.006 (6)	0.023 (6)	0.001 (6)
C32	0.065 (8)	0.088 (9)	0.072 (8)	0.018 (7)	0.027 (7)	0.007 (7)
C33	0.060 (7)	0.094 (9)	0.068 (8)	0.008 (7)	0.012 (6)	0.012 (7)
C34	0.074 (8)	0.081 (8)	0.064 (7)	0.015 (7)	0.033 (6)	0.003 (6)
C35	0.082 (8)	0.070 (8)	0.065 (7)	0.004 (7)	0.024 (7)	−0.012 (6)
C36	0.060 (6)	0.073 (8)	0.058 (7)	0.008 (6)	0.019 (5)	−0.004 (6)

### Geometric parameters (Å, °)

**Table:**

Cr1—O3	1.583 (7)	C6—C7	1.307 (12)
Cr1—O2	1.587 (7)	C6—H6	0.9300
Cr1—O1	1.614 (6)	C7—C8	1.440 (12)
Cr1—O4	1.753 (6)	C7—H7	0.9300
Cr2—O6	1.593 (7)	C8—C9	1.389 (12)
Cr2—O7	1.599 (6)	C8—C10	1.398 (13)
Cr2—O5	1.600 (6)	C10—C11	1.381 (13)
Cr2—O4	1.786 (6)	C10—H10	0.9300
Ni1—N3	2.064 (7)	C11—C12	1.370 (12)
Ni1—N2	2.072 (7)	C11—H11	0.9300
Ni1—N4	2.084 (7)	C12—H12	0.9300
Ni1—N1	2.088 (7)	C13—C14	1.411 (12)
Ni1—N6	2.094 (8)	C13—H13	0.9300
Ni1—N5	2.115 (7)	C14—C15	1.359 (13)
N1—C1	1.298 (11)	C14—H14	0.9300
N1—C5	1.346 (10)	C15—C16	1.375 (13)
N2—C12	1.310 (11)	C15—H15	0.9300
N2—C9	1.350 (10)	C16—C17	1.402 (12)
N3—C13	1.301 (10)	C16—C21	1.426 (14)
N3—C17	1.361 (11)	C17—C18	1.396 (12)
N4—C24	1.319 (11)	C18—C19	1.386 (12)
N4—C18	1.351 (11)	C19—C22	1.395 (13)
N5—C25	1.332 (11)	C19—C20	1.442 (13)
N5—C29	1.364 (11)	C20—C21	1.296 (13)
N6—C36	1.324 (11)	C20—H20	0.9300
N6—C30	1.391 (10)	C21—H21	0.9300
O8—H8C	0.8502	C22—C23	1.353 (13)
O8—H8D	0.8500	C22—H22	0.9300
O9—H9C	0.8500	C23—C24	1.407 (12)
O9—H9D	0.8501	C23—H23	0.9300
O9—H9A	0.8500	C24—H24	0.9300
O10—H10C	0.8502	C25—C26	1.425 (12)
O10—H10D	0.8500	C25—H25	0.9300
O11—H11C	0.8500	C26—C27	1.335 (13)
O11—H11D	0.8501	C26—H26	0.9300
O12—H12C	0.8500	C27—C28	1.388 (13)
O12—H12D	0.8500	C27—H27	0.9300
O12—H12A	0.8499	C28—C29	1.388 (12)
O13—H13C	0.8498	C28—C33	1.416 (13)
O13—H13D	0.8498	C29—C30	1.401 (12)
O14—H14C	0.8500	C30—C31	1.393 (12)
O14—H14D	0.8500	C31—C34	1.393 (13)
C1—C2	1.405 (12)	C31—C32	1.434 (13)
C1—H1	0.9300	C32—C33	1.298 (13)
C2—C3	1.342 (12)	C32—H32	0.9300
C2—H2	0.9300	C33—H33	0.9300
C3—C4	1.369 (12)	C34—C35	1.386 (12)
C3—H3	0.9300	C34—H34	0.9300
C4—C5	1.404 (11)	C35—C36	1.364 (12)
C4—C6	1.463 (12)	C35—H35	0.9300
C5—C9	1.428 (12)	C36—H36	0.9300
			
O3—Cr1—O2	108.4 (5)	C8—C9—C5	119.3 (8)
O3—Cr1—O1	109.5 (4)	C11—C10—C8	119.8 (10)
O2—Cr1—O1	111.4 (4)	C11—C10—H10	120.1
O3—Cr1—O4	109.7 (4)	C8—C10—H10	120.1
O2—Cr1—O4	109.0 (4)	C12—C11—C10	117.5 (10)
O1—Cr1—O4	108.9 (3)	C12—C11—H11	121.3
O6—Cr2—O7	109.6 (4)	C10—C11—H11	121.3
O6—Cr2—O5	110.7 (4)	N2—C12—C11	125.4 (9)
O7—Cr2—O5	108.1 (4)	N2—C12—H12	117.3
O6—Cr2—O4	107.1 (3)	C11—C12—H12	117.3
O7—Cr2—O4	111.3 (3)	N3—C13—C14	123.3 (10)
O5—Cr2—O4	110.1 (3)	N3—C13—H13	118.4
N3—Ni1—N2	98.5 (3)	C14—C13—H13	118.4
N3—Ni1—N4	79.7 (3)	C15—C14—C13	117.6 (10)
N2—Ni1—N4	95.3 (3)	C15—C14—H14	121.2
N3—Ni1—N1	95.5 (3)	C13—C14—H14	121.2
N2—Ni1—N1	79.1 (3)	C14—C15—C16	121.5 (10)
N4—Ni1—N1	172.1 (3)	C14—C15—H15	119.3
N3—Ni1—N6	91.9 (3)	C16—C15—H15	119.3
N2—Ni1—N6	168.5 (3)	C15—C16—C17	116.9 (10)
N4—Ni1—N6	91.5 (3)	C15—C16—C21	124.5 (11)
N1—Ni1—N6	95.0 (3)	C17—C16—C21	118.6 (10)
N3—Ni1—N5	168.9 (3)	N3—C17—C18	118.2 (8)
N2—Ni1—N5	91.5 (3)	N3—C17—C16	122.5 (9)
N4—Ni1—N5	94.7 (3)	C18—C17—C16	119.2 (9)
N1—Ni1—N5	91.1 (3)	N4—C18—C19	121.9 (9)
N6—Ni1—N5	78.6 (3)	N4—C18—C17	117.2 (9)
C1—N1—C5	118.2 (8)	C19—C18—C17	120.9 (10)
C1—N1—Ni1	129.1 (7)	C18—C19—C22	117.3 (10)
C5—N1—Ni1	112.7 (6)	C18—C19—C20	118.2 (10)
C12—N2—C9	116.9 (8)	C22—C19—C20	124.4 (10)
C12—N2—Ni1	129.9 (7)	C21—C20—C19	121.0 (11)
C9—N2—Ni1	112.6 (6)	C21—C20—H20	119.5
C13—N3—C17	118.2 (8)	C19—C20—H20	119.5
C13—N3—Ni1	129.6 (7)	C20—C21—C16	121.8 (11)
C17—N3—Ni1	112.0 (6)	C20—C21—H21	119.1
C24—N4—C18	118.9 (8)	C16—C21—H21	119.1
C24—N4—Ni1	128.9 (7)	C23—C22—C19	121.7 (10)
C18—N4—Ni1	112.3 (6)	C23—C22—H22	119.1
C25—N5—C29	118.3 (8)	C19—C22—H22	119.1
C25—N5—Ni1	128.4 (7)	C22—C23—C24	116.8 (10)
C29—N5—Ni1	113.3 (6)	C22—C23—H23	121.6
C36—N6—C30	117.0 (8)	C24—C23—H23	121.6
C36—N6—Ni1	129.8 (7)	N4—C24—C23	123.3 (10)
C30—N6—Ni1	113.2 (6)	N4—C24—H24	118.4
Cr1—O4—Cr2	131.4 (4)	C23—C24—H24	118.4
H8C—O8—H8D	108.7	N5—C25—C26	121.4 (10)
H9C—O9—H9D	108.3	N5—C25—H25	119.3
H9C—O9—H9A	109.9	C26—C25—H25	119.3
H9D—O9—H9A	109.9	C27—C26—C25	119.5 (10)
H10C—O10—H10D	108.6	C27—C26—H26	120.3
H11C—O11—H11D	107.4	C25—C26—H26	120.3
H12C—O12—H12D	108.8	C26—C27—C28	120.1 (10)
H12C—O12—H12A	110.6	C26—C27—H27	119.9
H12D—O12—H12A	110.6	C28—C27—H27	119.9
H13C—O13—H13D	108.5	C29—C28—C27	118.5 (10)
H14C—O14—H14D	108.3	C29—C28—C33	118.1 (10)
N1—C1—C2	123.0 (9)	C27—C28—C33	123.4 (11)
N1—C1—H1	118.5	N5—C29—C28	122.1 (9)
C2—C1—H1	118.5	N5—C29—C30	117.6 (9)
C3—C2—C1	118.5 (10)	C28—C29—C30	120.3 (10)
C3—C2—H2	120.7	N6—C30—C31	122.6 (9)
C1—C2—H2	120.7	N6—C30—C29	117.1 (9)
C2—C3—C4	120.6 (9)	C31—C30—C29	120.3 (10)
C2—C3—H3	119.7	C34—C31—C30	117.5 (10)
C4—C3—H3	119.7	C34—C31—C32	125.0 (11)
C3—C4—C5	117.3 (9)	C30—C31—C32	117.4 (10)
C3—C4—C6	125.2 (9)	C33—C32—C31	121.7 (11)
C5—C4—C6	117.4 (10)	C33—C32—H32	119.1
N1—C5—C4	122.3 (9)	C31—C32—H32	119.1
N1—C5—C9	116.7 (8)	C32—C33—C28	122.1 (11)
C4—C5—C9	120.9 (9)	C32—C33—H33	118.9
C7—C6—C4	121.0 (10)	C28—C33—H33	118.9
C7—C6—H6	119.5	C35—C34—C31	119.5 (10)
C4—C6—H6	119.5	C35—C34—H34	120.3
C6—C7—C8	122.3 (10)	C31—C34—H34	120.3
C6—C7—H7	118.9	C36—C35—C34	119.5 (10)
C8—C7—H7	118.9	C36—C35—H35	120.3
C9—C8—C10	117.0 (9)	C34—C35—H35	120.3
C9—C8—C7	119.0 (9)	N6—C36—C35	123.9 (10)
C10—C8—C7	124.0 (10)	N6—C36—H36	118.0
N2—C9—C8	123.5 (9)	C35—C36—H36	118.0
N2—C9—C5	117.1 (8)		
			
N3—Ni1—N1—C1	−74.9 (8)	C10—C8—C9—N2	1.3 (13)
N2—Ni1—N1—C1	−172.5 (8)	C7—C8—C9—N2	−177.8 (8)
N4—Ni1—N1—C1	−127 (2)	C10—C8—C9—C5	178.2 (8)
N6—Ni1—N1—C1	17.4 (8)	C7—C8—C9—C5	−0.9 (13)
N5—Ni1—N1—C1	96.1 (8)	N1—C5—C9—N2	−3.0 (11)
N3—Ni1—N1—C5	107.9 (6)	C4—C5—C9—N2	176.4 (7)
N2—Ni1—N1—C5	10.3 (5)	N1—C5—C9—C8	179.9 (7)
N4—Ni1—N1—C5	56 (2)	C4—C5—C9—C8	−0.7 (13)
N6—Ni1—N1—C5	−159.8 (6)	C9—C8—C10—C11	−1.9 (13)
N5—Ni1—N1—C5	−81.1 (6)	C7—C8—C10—C11	177.2 (9)
N3—Ni1—N2—C12	83.5 (8)	C8—C10—C11—C12	0.5 (14)
N4—Ni1—N2—C12	3.2 (8)	C9—N2—C12—C11	−2.5 (13)
N1—Ni1—N2—C12	177.5 (8)	Ni1—N2—C12—C11	167.9 (7)
N6—Ni1—N2—C12	−122.6 (15)	C10—C11—C12—N2	1.9 (15)
N5—Ni1—N2—C12	−91.7 (8)	C17—N3—C13—C14	−0.6 (14)
N3—Ni1—N2—C9	−105.8 (6)	Ni1—N3—C13—C14	173.6 (7)
N4—Ni1—N2—C9	173.9 (6)	N3—C13—C14—C15	−0.9 (15)
N1—Ni1—N2—C9	−11.8 (6)	C13—C14—C15—C16	1.9 (15)
N6—Ni1—N2—C9	48.2 (17)	C14—C15—C16—C17	−1.4 (15)
N5—Ni1—N2—C9	79.0 (6)	C14—C15—C16—C21	−179.8 (10)
N2—Ni1—N3—C13	84.9 (8)	C13—N3—C17—C18	−178.6 (8)
N4—Ni1—N3—C13	178.7 (8)	Ni1—N3—C17—C18	6.3 (10)
N1—Ni1—N3—C13	5.1 (8)	C13—N3—C17—C16	1.1 (13)
N6—Ni1—N3—C13	−90.1 (8)	Ni1—N3—C17—C16	−174.0 (7)
N5—Ni1—N3—C13	−121.0 (16)	C15—C16—C17—N3	−0.2 (14)
N2—Ni1—N3—C17	−100.7 (6)	C21—C16—C17—N3	178.3 (9)
N4—Ni1—N3—C17	−6.8 (6)	C15—C16—C17—C18	179.5 (9)
N1—Ni1—N3—C17	179.5 (6)	C21—C16—C17—C18	−2.0 (14)
N6—Ni1—N3—C17	84.3 (6)	C24—N4—C18—C19	−4.1 (13)
N5—Ni1—N3—C17	53.5 (19)	Ni1—N4—C18—C19	175.1 (7)
N3—Ni1—N4—C24	−174.3 (9)	C24—N4—C18—C17	175.5 (8)
N2—Ni1—N4—C24	−76.7 (8)	Ni1—N4—C18—C17	−5.3 (10)
N1—Ni1—N4—C24	−122 (2)	N3—C17—C18—N4	−0.6 (13)
N6—Ni1—N4—C24	94.0 (8)	C16—C17—C18—N4	179.7 (8)
N5—Ni1—N4—C24	15.3 (8)	N3—C17—C18—C19	179.0 (8)
N3—Ni1—N4—C18	6.6 (6)	C16—C17—C18—C19	−0.7 (14)
N2—Ni1—N4—C18	104.2 (6)	N4—C18—C19—C22	4.7 (14)
N1—Ni1—N4—C18	59 (3)	C17—C18—C19—C22	−174.8 (9)
N6—Ni1—N4—C18	−85.1 (6)	N4—C18—C19—C20	−178.9 (8)
N5—Ni1—N4—C18	−163.8 (6)	C17—C18—C19—C20	1.6 (14)
N3—Ni1—N5—C25	−151.4 (15)	C18—C19—C20—C21	0.5 (15)
N2—Ni1—N5—C25	3.0 (8)	C22—C19—C20—C21	176.6 (10)
N4—Ni1—N5—C25	−92.4 (8)	C19—C20—C21—C16	−3.3 (17)
N1—Ni1—N5—C25	82.2 (8)	C15—C16—C21—C20	−177.5 (10)
N6—Ni1—N5—C25	177.0 (8)	C17—C16—C21—C20	4.1 (16)
N3—Ni1—N5—C29	28.3 (19)	C18—C19—C22—C23	−2.0 (15)
N2—Ni1—N5—C29	−177.3 (6)	C20—C19—C22—C23	−178.2 (9)
N4—Ni1—N5—C29	87.3 (6)	C19—C22—C23—C24	−1.1 (15)
N1—Ni1—N5—C29	−98.1 (6)	C18—N4—C24—C23	0.6 (14)
N6—Ni1—N5—C29	−3.3 (6)	Ni1—N4—C24—C23	−178.4 (7)
N3—Ni1—N6—C36	8.1 (8)	C22—C23—C24—N4	1.9 (15)
N2—Ni1—N6—C36	−146.1 (13)	C29—N5—C25—C26	1.1 (13)
N4—Ni1—N6—C36	87.8 (8)	Ni1—N5—C25—C26	−179.2 (6)
N1—Ni1—N6—C36	−87.6 (8)	N5—C25—C26—C27	−1.9 (14)
N5—Ni1—N6—C36	−177.7 (8)	C25—C26—C27—C28	0.9 (14)
N3—Ni1—N6—C30	−170.3 (6)	C26—C27—C28—C29	0.7 (14)
N2—Ni1—N6—C30	35.4 (18)	C26—C27—C28—C33	179.3 (9)
N4—Ni1—N6—C30	−90.6 (6)	C25—N5—C29—C28	0.6 (12)
N1—Ni1—N6—C30	94.0 (6)	Ni1—N5—C29—C28	−179.2 (6)
N5—Ni1—N6—C30	3.9 (6)	C25—N5—C29—C30	−178.1 (8)
O3—Cr1—O4—Cr2	27.0 (7)	Ni1—N5—C29—C30	2.1 (9)
O2—Cr1—O4—Cr2	−91.5 (6)	C27—C28—C29—N5	−1.5 (13)
O1—Cr1—O4—Cr2	146.8 (5)	C33—C28—C29—N5	179.9 (8)
O6—Cr2—O4—Cr1	159.5 (5)	C27—C28—C29—C30	177.2 (8)
O7—Cr2—O4—Cr1	−80.7 (6)	C33—C28—C29—C30	−1.5 (13)
O5—Cr2—O4—Cr1	39.1 (6)	C36—N6—C30—C31	−0.6 (12)
C5—N1—C1—C2	2.4 (13)	Ni1—N6—C30—C31	178.0 (7)
Ni1—N1—C1—C2	−174.6 (6)	C36—N6—C30—C29	177.3 (8)
N1—C1—C2—C3	−0.2 (14)	Ni1—N6—C30—C29	−4.1 (9)
C1—C2—C3—C4	−0.3 (14)	N5—C29—C30—N6	1.3 (11)
C2—C3—C4—C5	−1.4 (13)	C28—C29—C30—N6	−177.4 (7)
C2—C3—C4—C6	−178.0 (8)	N5—C29—C30—C31	179.2 (8)
C1—N1—C5—C4	−4.3 (12)	C28—C29—C30—C31	0.5 (13)
Ni1—N1—C5—C4	173.3 (6)	N6—C30—C31—C34	0.1 (13)
C1—N1—C5—C9	175.2 (8)	C29—C30—C31—C34	−177.8 (8)
Ni1—N1—C5—C9	−7.3 (9)	N6—C30—C31—C32	178.5 (8)
C3—C4—C5—N1	3.8 (13)	C29—C30—C31—C32	0.7 (13)
C6—C4—C5—N1	−179.4 (7)	C34—C31—C32—C33	177.4 (10)
C3—C4—C5—C9	−175.6 (8)	C30—C31—C32—C33	−1.0 (14)
C6—C4—C5—C9	1.2 (12)	C31—C32—C33—C28	0.0 (16)
C3—C4—C6—C7	176.4 (9)	C29—C28—C33—C32	1.2 (15)
C5—C4—C6—C7	−0.1 (13)	C27—C28—C33—C32	−177.3 (10)
C4—C6—C7—C8	−1.5 (15)	C30—C31—C34—C35	1.6 (14)
C6—C7—C8—C9	2.0 (14)	C32—C31—C34—C35	−176.8 (9)
C6—C7—C8—C10	−177.0 (9)	C31—C34—C35—C36	−2.7 (15)
C12—N2—C9—C8	0.8 (13)	C30—N6—C36—C35	−0.5 (13)
Ni1—N2—C9—C8	−171.3 (7)	Ni1—N6—C36—C35	−178.9 (7)
C12—N2—C9—C5	−176.2 (7)	C34—C35—C36—N6	2.2 (15)
Ni1—N2—C9—C5	11.8 (9)		

### Hydrogen-bond geometry (Å, °)

**Table:**

*D*—H···*A*	*D*—H	H···*A*	*D*···*A*	*D*—H···*A*
O14—H14D···O10^i^	0.85	2.06	2.91 (3)	177
O14—H14C···O10^ii^	0.85	1.75	2.60 (3)	176
O13—H13D···O14	0.85	1.76	2.61 (3)	179
O13—H13C···O2^iii^	0.85	1.98	2.83 (2)	176
O12—H12D···O9^iv^	0.85	2.16	2.99 (2)	165
O12—H12C···O14	0.85	2.11	2.93 (3)	165
O10—H10D···O11	0.85	1.93	2.78 (3)	171
O10—H10C···O1	0.85	1.91	2.751 (17)	172
O9—H9A···O12^v^	0.85	2.24	2.99 (2)	147
O9—H9D···O9^vi^	0.85	1.94	2.78 (3)	176
O9—H9C···O8	0.85	2.11	2.962 (17)	177
O8—H8D···O11^vii^	0.85	1.92	2.76 (2)	167
O8—H8C···O6	0.85	2.00	2.836 (11)	168

Symmetry codes: (i) −*x*+3/2, −*y*+1/2, −*z*+1; (ii) *x*−1/2, *y*−1/2, *z*; (iii) −*x*+3/2, *y*−1/2, −*z*+3/2; (iv) *x*, −*y*+1, *z*−1/2; (v) *x*, −*y*+1, *z*+1/2; (vi) −*x*+1, *y*, −*z*+3/2; (vii) −*x*+3/2, −*y*+3/2, −*z*+1.
